# The Association Between Depression and Obesity Among Adults in the Eastern Province, Saudi Arabia

**DOI:** 10.7759/cureus.18794

**Published:** 2021-10-14

**Authors:** Fatimah H Almarhoon, Khadijaa A Almubarak, Zahra A Alramdhan, Rafah S Albagshi, Jannah K Alotayriz, Abdullah H Alqahtani

**Affiliations:** 1 Psychiatry, Imam Abdulrahman Bin Faisal University, Khobar, SAU

**Keywords:** body mass index, patient health questionnaire (phq-9), saudi arabia, eastern province, adult, obesity, depression

## Abstract

Background: Depression is a primary cause of disability-adjusted life years lost globally. It is a common mental disorder with roughly more than 264 million adults affected. Obesity is another major health problem affecting more than 650 million adults worldwide. The presence of depression and obesity, along with each other, is associated with more negative health outcomes.

Objectives: To explore the correlation between depression and obesity among adults in the Eastern Province of Saudi Arabia and analyze this association with other variables, including patients' demographics, body mass index (BMI), and presence of chronic and psychiatric illness.

Method: A cross-sectional study was done in the Eastern Province, Saudi Arabia. A total number of 711 participants were enrolled. Arabic version of Patient Health Questionnaire 9 (PHQ-9) was used. Body mass index (BMI) scores were used to classify participants into underweight, average weight, overweight, and obese.

Result: It was found that 41.7% of the obese participants have moderate to severe depression, and this result was statistically significant (P = 0.027, 95% CI 1.69-1.98). The prevalence was more marked among young participants (P = 0.001). Other variables such as marital status, the presence of a chronic illness, psychiatric disorders, regular intake of medications, effect of depressive symptoms on daily activity, and the number of years diagnosed with obesity and depression all showed a statistically significant association in the presence of comorbidity of obesity and depression (P < 0.05).

Conclusion: The association between depression and obesity is most prominent in young adults aged between 18 to 25 years (11.2%), being single (12.8%), having a BMI of 30 or more for 10 years or more (45.4%), the presence of associated chronic illnesses (17.6%), the presence of associated psychiatric disorder (18.3%) and intake of regular medications (18.3%). Depression and obesity are major health challenges worldwide. Many studies were done to assess the relationship between obesity and depression, but only a few were conducted in Saudi Arabia. This study was done to investigate this relationship. It will help raise awareness about the comorbidity of depression and obesity to address preventative and therapeutic measures.

## Introduction

World Health Organization (WHO) stated that depression in both genders is a primary cause of disability-adjusted life years lost globally. Also, it is a common mental disorder affecting more than 264 million people. Depressive episodes are mainly present with sadness, loss of interest, and lack of pleasure [1]. Another major health problem in modern societies is obesity, affecting more than 650 million adults worldwide. Researchers mainly use body mass index (BMI) to detect and classify adults as overweight and obese [2].

The presence of depression and obesity along with each other is associated with more negative health outcomes than either one of these health problems alone. Moreover, both conditions showed association with other comorbidities leading to a more significant health impact [3]. Obesity causes adipose tissue alteration and increases gut permeability, which induces proinflammatory cytokines. Cytokines generate a low-grade chronic systemic inflammatory response that eventually could reach the brain via multiple routes, including the vagus nerve. That leads to neuroinflammation disturbing the brain's function and neurotransmitters, including dopamine, serotonin, and glutamate [4].

This research explores the correlation between depression and obesity among adults in Eastern Province, Saudi Arabia. A cross-sectionally designed and population-based questionnaire was distributed and analyzed to answer the relationship between these critical health issues. Many studies have tried to assess the relationship between depression and obesity, but not all these studies evaluated the relationship considering variables and confounders that may affect the findings. In Saudi Arabia, studies in this area are scarce. Also, many studies attempted to explore the pathophysiology of these comorbidities and why they occur. 

## Materials and methods

**Figure 1:**
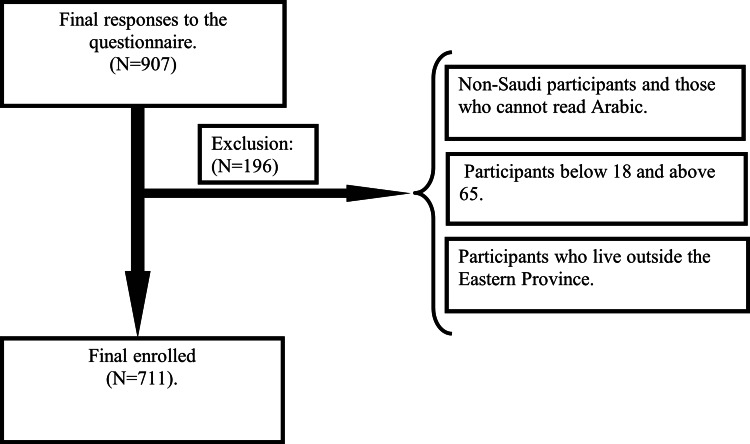
Study participant selection flow chart

This cross-sectional study was conducted after receiving approval from the Imam Abdulrahman Bin Faisal University Institutional Review Board (approval number: IRB-UGS-2020-01-284). Participants were male and female Saudis aged between 18-65 years residing in the Eastern Province. The information was collected through an online questionnaire after obtaining informed consent. This study was conducted during the period from October 2020 to February 2021. The calculated sample size was 384 participants, which was determined based on power calculations to ensure that the study would detect statistically significant differences [5]. Therefore, the sample size was derived by computing the minimum sample size required for accuracy in estimating proportions by considering the normal standard deviation set at 95% confidence level (1.96), percentage picking a choice or response (50% = 0.5), and the confidence interval. Inclusion criteria were age between 18-65 years, Saudi nationality, and residence in Eastern Province. Exclusion criteria were age below 18 or above 65 years, non-Saudi nationality, and living outside Eastern Province (Figure 1).

Data was collected through an electronic survey. Demographic data included: age, sex, education, occupation, marital status, and socioeconomic status. Also, data were collected regarding physical and mental illness and medication intake. An Arabic version of Patient Health Questionnaire 9 (PHQ-9) was used. It is a valid and reliable assessment tool for depression in both clinical and research settings [6]. The participants were asked to mention their height in centimeters and weight in kilograms and using this information, the BMI of participants was calculated automatically via a programmed formula within the online questioner, leading to accurate results. The sample population was categorized according to the BMI into underweight, normal weight, overweight, and obese. Furthermore, it was classified according to the presence or absence of depression. The analysis focused on three subgroups: obesity with depression (Group A), obesity without depression (Group B), depression without obesity (Group C). These three groups were compared to each other to find if there are statistically significant differences in their characteristics [7]. Statistical analysis was done using SPSS Software for Windows, version 26.0 (IBM Corp., Armonk, NY) and VassarStats website (Lowry R, VassarStats: Website for Statistical Computation. http://vassarstats.net/). 

## Results

Various demographic characteristics of 711 participants are shown in Table 1. In terms of gender, 177 (24.9%) were males, and 534 (75.1%) were females. In terms of the age distribution, 47.7% of participants were between the age of 18-25 years, 43.7% belonged to the age range of 26-45 years, and only 8.6% were above 45 years of age. Out of the total, 45.9% of participants were single, 51.9% were married, and the rest had other marital statuses. The participants had a different level of education, but most of them were at the university level or above, accounting for 79.5% of all participants. Also, the participants had financial differences, as 47.5% of them had an average monthly income of less than 10,000 Saudi Riyals (SAR), and 52.5% had 10,000 SAR or above. Most of them had less than five family members who benefit from that income. After studying the BMI in these participants, it was revealed that most of them had average weight, which accounts for 35.7%, or overweight that accounts for 30.5%. Moreover, it also showed that 26.4% were obese, and 41.2% of them had been diagnosed with obesity ten years ago or more. Most participants did not suffer from chronic diseases, which accounts for 81.6% of the total, and 84.7% did not take any regular medication. The result from the PHQ-9 depression screening on those participants showed that 65.2% of the total participants had no or mild depression, but 34.8% having moderate to severe depression. Only 8.1% of them have depressive symptoms that affect their daily life activities. Participants diagnosed with psychiatric disorders less than ten years ago were 34.4%, and those who did not know or remember when they were diagnosed constituted 60.3% of the total participants. 

**Table 1:** Characteristics of the participants

Participant's characteristics	N (%)
Gender	
Male	177 (24.9)
Female	534 (75.1)
Age, Years	
18 to 25	339 (47.7)
26 to 45	311 (43.7)
More than 45	61 (8.6)
Marital status	
Single	326 (45.9)
Married	369 (51.9)
Other (Divorced or Widow)	16 (2.2)
Average monthly income	
Less than 10,000	338 (47.5)
10,000 or more	373 (52.5)
Number of family members benefiting from the income	
5 or Less	436 (61.3)
More than 5	275 (38.7)
Educational level	
High school or below	146 (20.5)
University or above	565 (79.5)
Body mass index	
Under weight (less than 18.5)	53 (7.4)
Normal weight (18.5 to 24.9)	254 (35.7)
Overweight (25 to 29.9)	217 (30.5)
Obese (30 or more)	187 (26.4)
Number of years with BMI of 30 or more	
Less than 10 years	73 (39.1)
10 years or more	77 (41.2)
Not known date	37 (19.7)
The presence of chronic illnesses	
No	552 (77.6)
Yes	159 (22.4)
The presence of psychiatric disorders	
No	580 (81.6)
Yes	131 (18.4)
Intake of regular medication	
No	602 (84.7)
Yes	109 (15.3)
The result from the PHQ-9 depression screening	
No depression or mild depression	464 (65.2)
Depression moderate or more (cut-off point of ≥ 10)	247 (34.8)
Effect of the depressive symptoms on daily life	
No or mild effect	654 (91.9)
Yes (moderate or more)	57 (8.1)
Number of years with the diagnosis of psychiatric disorder	
Less than 10 years	45 (34.4)
10 years or more	7 (5.3)
Not known date	79 (60.3)

Table 2 shows the incidence of depression according to the variables to demonstrate the relationship between depression and each variable. It shows that 35% of females and 33.9% of males have a moderate to severe risk of developing depression, according to the PHQ-9 questionnaire. The result showed that the risk of depression was more common in the young adult age group from 18-25 years old, at a rate of 46%, and less common in participants who are older than 45 years old, at a rate of 11.5%. Depression was quite prominent in divorced or widowed participants by 31.3%. The table shows that 41.7% of the obese participants have a moderate to severe risk of depression, but interestingly, it also shows that 43.4% of the total underweight participants have the same risk of depression. Out of the total people diagnosed with obesity ten years ago or more, 41% suffered from depression; out of those diagnosed with obesity less than ten years ago, 30% were depressed; and only 10% of obese and depressed patients did not know their obesity diagnosis date. Participants who suffer from depression along with other chronic diseases constitute 42.8%, and 32.4% of depression patients did not suffer from any chronic diseases. Out of the total patients who suffered from depression, 60% had other psychiatric disorders, but 29% did not, and 47.7% take medication, but 32.4% did not. Out of all the depressed participants, 89.5% had moderate or severe symptoms that interfere with their daily life activities. Out of the total, 71.4% of the participants who suffer from depression were diagnosed for ten years or more, 71.1% were diagnosed less than ten years ago, and only 38% do not know the exact date of the diagnosis. The table also shows other characteristics and variables like the average monthly income, numbers of the family that benefits from that income, and the educational level. However, according to the p-value, they were statistically insignificant. 

**Table 2:** Relationship between depression and other characteristics of participants N = number of participant; OR = odds ratio; CI = confidence interval

Participants characteristics	N (%)	Depression cases (%)	OR (95% CI)	p-value
Gender				0.786
Male	177 (24.9)	60 (33.9)	1.68 (1.54-1.82)	
Female	534 (75.1)	187 (35)	1.70 (1.62-1.78)	
Age, Years				0.0001
18 to 25	339 (47.7)	156 (46)	1.92 (1.81-2.03)	
26 to 45	311 (43.7)	84 (27)	1.54 (1.44-1.64)	
More than 45	61 (8.6)	7 (11.5)	1.23 (1.06-1.39)	
Marital status				0.0001
Single	326 (45.9)	141 (43.3)	1.87 (1.76-1.97)	
Married	369 (51.9)	101 (27.4)	1.55 (1.46-1.64)	
Other (Divorced or Widow)	16 (2.2)	5 (31.3)	1.63 (1.11-2.14)	
Average monthly income				0.803
Less than 10,000	338 (47.5)	119 (35.2)	1.70 (1.60-1.81)	
10,000 or more	373 (52.5)	128 (34.3)	1.69 (1.59-1.78)	
Number of family members benefiting from the income				0.575
Less than 5	436 (61.3)	148 (34)	1.68 (1.59-1.77)	
5 or more	275 (38.7)	99 (36)	1.72 (1.61-1.83)	
Educational level				0.522
High school or below	146 (20.5)	54 (37)	1.74 (1.58-1.90)	
University or above	565 (79.5)	193 (34.2)	1.68 (1.60-1.76)	
Body mass index				0.027
Under weight (less than 18.5)	53 (7.5)	23 (43.4)	1.87 (1.59-2.14)	
Normal weight (18.5 to 24.9)	254 (35.7)	75 (29.5)	1.59 (1.48-1.70)	
Overweight (25 to 29.9)	217 (30.5)	71 (32.7)	1.65 (1.53-1.78)	
Obese (30 or more)	187 (26.4)	78 (41.7)	1.83 (1.69-1.98)	
Number of years with BMI of 30 or more				0.046
Less than 10 years	73 (39.1)	30 (41.1)	1.82 (1.59-2.05)	
10 years or more	77 (41.2)	36 (46.8)	1.94 (1.71-2.16)	
Not known date	37 (19.7)	12 (32.4)	1.73 (1.38-2.07)	
The presence of chronic illnesses				0.013
No	552 (77.6)	179 (32.4)	1.65 (1.57-1.73)	
Yes	159 (22.4)	68 (42.8)	1.86 (1.70-2.02)	
The presence of psychiatric disorders				0.0001
No	580 (81.6)	168 (29)	1.58 (1.51-1.65)	
Yes	131 (18.4)	79 (60.3)	2.21 (2.04-2.38)	
Intake of regular medication				0.002
No	602 (84.7)	195 (32.4)	1.65 (1.57-1.72)	
Yes	109 (15.3)	52 (47.7)	1.95 (1.76-2.14)	
Effect of the depressive symptoms on daily life				0.0001
No or mild effect	654 (91.9)	196 (30)	1.60 (1.53-1.67)	
Yes (moderate or more)	57 (8.1)	51 (89.5)	2.79 (2.63-2.95)	
Number of years with the diagnosis of psychiatric disorder				0.0001
Less than 10 years	45 (34.4)	32 (71.1)	2.42 (2.15-2.70)	
10 years or more	7 (5.3)	5 (71.4)	2.43 (1.53-3.33)	
Not known date	79 (60.3)	30 (38)	1.74 (1.53-1.96)	

Table 3 shows the incidence of obesity corresponding to different study variables to demonstrate the relationship between obesity and each variable. Out of the total male participants, 36.7% were obese and out of the total female participants, 22.8% were obese. Obesity was more prevalent among participants aged 45 years or more, compared to the younger age groups. It was 41%, 30.2%, and 20.1% for the age groups 45 years or more, 26-45 years, and 18-25 years, respectively. There was a difference in the prevalence between single (23.3%), married (28.7), and divorced or widowed participants (31.3%). The presence of chronic illness has affected the incidence of obesity, as 36.5% of participants with chronic illness had obesity while only 23.2% of participants who denied the existence of any chronic disease were obese. A higher incidence of obesity (40.4%) was noted in people with moderate to severe depression in comparison to only 23.8% of cases of obesity observed in people with mild depressive symptoms or without any symptoms of depression. The association between the presence of psychiatric disorders in general and the probability of having obesity was statistically insignificant (p=0.756). Participants suffering from psychiatric disorders for less than ten years were at a higher risk of developing obesity (33.3%), while obesity was noted only in 28.6% of participants who were diagnosed with psychiatric disorders for ten years or more, this observation was statistically significant (p=0.007). Individuals who take regular medications showed a prevalence of 40.4%, which is higher than those who do not take medications regularly as only 23.8% of them were obese.

**Table 3:** Relationship between obesity and other characteristics of the participants N = Number of participants; OR = Odds ratio (OR)

Participant's characteristics	N (%)	Obesity cases (%)	OR (95% CI)	p-value
Gender				0.001
Male	177 (24.9)	65 (36.7)	2.99(2.85-3.13)	
Female	534 (75.1)	122 (22.8)	2.68(2.6.-2.76)	
Age, Years				0.000
18 to 25	339 (47.7)	68 (20.1)	2.50(2.40-2.60)	
26 to 45	311 (43.7)	94 (30.2)	2.96(2.87-3.05)	
More than 45	61 (8.6)	25 (41)	3.16(2.96-3.37)	
Marital status				0.000
Single	326 (45.9)	76 (23.3)	2.59(2.48-2.69)	
Married	369 (51.9)	106 (28.7)	2.89(2.80-2.98)	
Other (Divorced or Widow)	16 (2.2)	5 (31.3)	3.06(2.65-3.47)	
Average monthly income				0.083
Less than 10,000	338 (47.5)	98 (29)	2.84(2.74-2.94)	
10,000 or more	373 (52.5)	89 (23.9)	2.68(2.58-2.77)	
Number of family members benefiting from the income				0.125
Less than 5	436 (61.3)	105 (24.1)	2.73(2.65-2,82)	
5 or more	275 (38.7)	82 (29.9)	2.79(2.68-2.91)	
Educational level				0.550
High school or below	146 (20.5)	44 (30.1)	2.85(2.70-3.00)	
University or above	565 (79.5)	143 (25.3)	2.73(2.66-2.81)	
Number of years with BMI of 30 or more				0.000
Less than 10 years	73 (39.1)	73 (100)	4.00(4.00-4.00)	
10 years or more	77 (41.2)	76 (98.7)	3.99(3.96-4.01)	
Not known date	37 (19.7)	37 (100)	4.00(4.00-4.00)	
The presence of chronic illnesses				0.001
No	552 (77.6)	128 (23.2)	2.69(2.62-2.77)	
Yes	159 (22.4)	58 (36.5)	2.98(2.83-3.13)	
The presence of psychiatric disorders				0.756
No	580 (81.6)	148 (25.5)	2.74(2.66-2.82)	
Yes	131 (18.4)	39 (29.8)	2.83(2.67-2.99)	
Intake of regular medication				0.004
No	602 (84.7)	143 (23.8)	2.71(2.64-2.79)	
Yes	109 (15.3)	44 (40.4)	2.99(2.81-3.27)	
The result from the PHQ-9 depression screening				0.027
No depression or mild depression	464 (65.2)	109 (23.5)	2.72(2.64-2.80)	
Depression moderate or more (cut-off point of ≥ 10)	247 (34.8)	78 (31.6)	2.83(2.70-2.95)	
Effect of the depressive symptoms on daily life				0.095
No or mild effect	654 (91.9)	164 (25.1)	2.74(2.66-2.81)	
Yes (moderate or more)	57 (8.1)	23 (40.4)	3.00(2.74-3.26)	
Number of years with the diagnosis of psychiatric disorder				0.007
Less than 10 years	45 (34.4)	15 (33.3)	2.62(2.30-2.94)	
10 years or more	7 (5.3)	2 (28.6)	3.00(2.24-3.76)	
Not known date	79 (60.3)	20 (25.3)	2.81(2.61-3.2)	

Table 4 shows the number and percentage of cases with the concurrence of depression and obesity and their association with other study variables. The association was more pronounced among males (13.5%) compared to females (10.1%), which was not statistically significant (p=0.786). The prevalence among each age group was 11.2%, 9.1%, 9.8% for 18-25 years, 26-45 years, and 45 or more age groups. Single participants reported higher concurrence of depression and obesity, which was 12.8%, while married participants had 9.4%, widowed and divorced had 6.25%. Among the participants with chronic illness, 17.6% were both obese and depressed compared to 9.1% of healthy participants with obesity and depression. While checking for a psychiatric disorder, 18.3% of obese and depressed participants were diagnosed with a psychiatric issue, while 9.3% were free of mental illness. That means that having both increases the risk of a co-existing psychiatric disorder. The number of years with the disorder also had affected the association. Psychiatric patients diagnosed less than ten years ago had 22.2%, those who were diagnosed ten years or more ago had 14.2%, and those diagnosed for an unknown number of years had a 10.9% prevalence for concurrence of depression and obesity. Out of the total patients on regular medication, 18.3% of participants were obese and depressed whereas only 9.6% of patients not on regular medication were obese and depressed, which means that their concurrence can raise the risk of taking regular medications. The studied association with the concurrence of depression and obesity is also noticeable among participants whose depressive symptoms affect their daily lives moderate to severe (38.5%) compared to those who were mildly affected or were not affected (8.5%). Having obesity for more than ten years increases the risk of having comorbid depression; participants who are obese for more than ten years (45.4%) were affected more than those having obesity for less than ten years (41.1%).

**Table 4:** Relationship of obesity and depression concurrence with other characteristics of the participants N = Number of participants

Participant's characteristics	N (%)	Cases with both Obesity and Depression (%)	p-value
Gender			0.786
Male	177 (24.9)	24 (13.5)	
Female	534 (75.1)	54 (10.1)	
Age, Years			0.00
18 to 25	339 (47.7)	38 (11.2)	
26 to 45	311 (43.7)	34 (9.1)	
More than 45	61 (8.6)	6 (9.8)	
Marital status			0.00
Single	326 (45.9)	42 (12.8)	
Married	369 (51.9)	35 (9.4)	
Other (Divorced or Widow)	16 (2.2)	1 (6.25)	
Average monthly income			0.803
Less than 10,000	338 (47.5)	42 (12.4)	
10,000 or more	373 (52.5)	36 (9.6)	
Number of family members benefiting from the income			0.575
Less than 5	436 (61.3)	44 (10.1)	
5 or more	275 (38.7)	34 (12.3)	
Educational level			0.522
High school or below	146 (20.5)	18 (12.3)	
University or above	565 (79.5)	60 (10.6)	
Number of years with BMI of 30 or more			0.046
Less than 10 years	73 (39.1)	30 (41.1)	
10 years or more	77 (41.2)	35 (45.4)	
Not known date	37 (19.7)	13 (35.1)	
The presence of chronic illnesses			0.013
No	552 (77.6)	50 (9.1)	
Yes	159 (22.4)	28 (17.6)	
The presence of psychiatric disorders			0.00
No	580 (81.6)	54 (9.3)	
Yes	131 (18.4)	24 (18.3)	
Intake of regular medication			0.002
No	602 (84.7)	58 (9.6)	
Yes	109 (15.3)	20 (18.3)	
Effect of the depressive symptoms on daily life			0.00
No or mild effect	654 (91.9)	56 (8.5)	
Yes (moderate or more)	57 (8.1)	22 (38.5)	
Number of years with the diagnosis of psychiatric disorder			0.00
Less than 10 years	45 (34.4)	10 (22.2)	
10 years or more	7 (5.3)	1 (14.2)	
Not known date	79 (60.3)	8 (10.9)	

## Discussion

**Figure 2:**
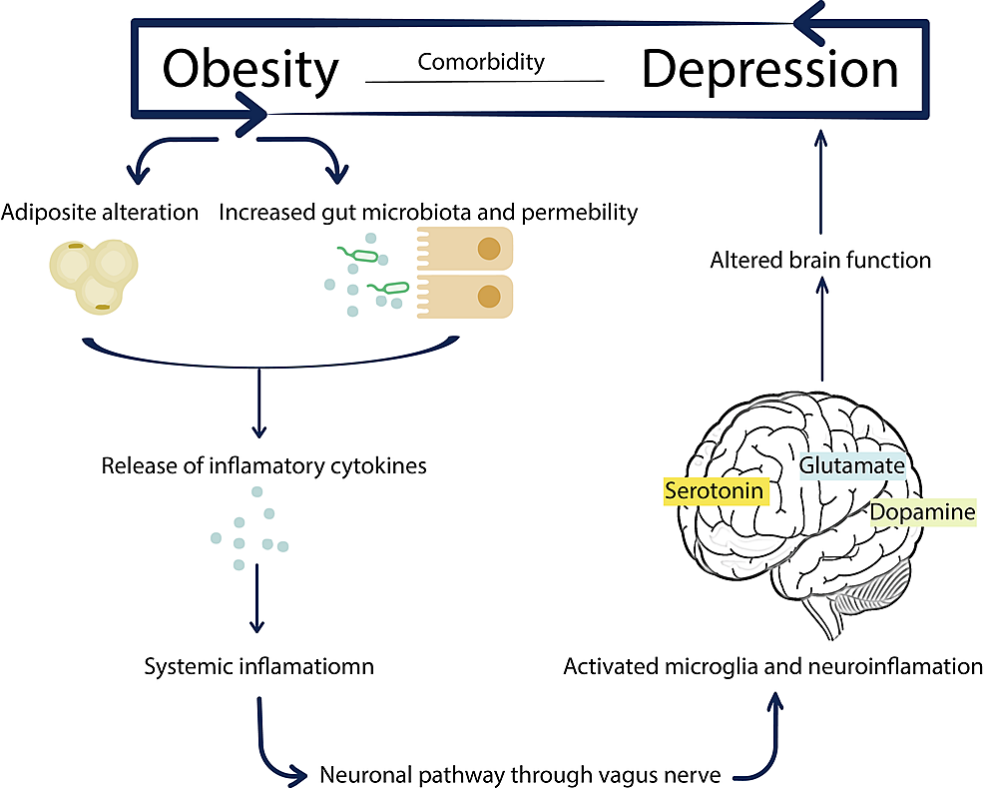
Pathophysiology of the comorbidity between obesity and depression

As mentioned earlier, the possible pathophysiology of these comorbidities is that obesity causes adipose tissue alteration inducing proinflammatory cytokines (Figure 2). These cytokines generate a systemic inflammatory response that could reach the brain via multiple routes leading to neuroinflammation that disturbs the brain's function and neurotransmitters [4]. 

The majority of the study participants were females (75.1%). This result may be due to how differently females and males use the internet. Men are less likely to interact socially compared to women. Moreover, women are more likely to engage in online activities that involve communication and knowledge exchange, resulting in variation in online survey response rate [8]. More than 90% of participants were aged 45 years or less. This result can be explained by the fact that most of Saudi Arabia’s population (72%) is between 15 to 64 years of age [9].

We observed high rates of being overweight or obese among the participants. One-third of participants were overweight, and more than a quarter was obese. These results were similar to those reported by other studies. Alshahrani et al., in Abha, Saudi Arabia, reported that the prevalence of being overweight or obese among secondary school males were (44.2% and 38.4%, respectively) [10]. However, a recent study among male students in Saudi Arabia reported a lower prevalence of being overweight or obese, 21.8% and 15.7%, respectively [11]. Simon et al., in the United States, reported that the prevalence of obesity in middle-aged women (40-65 years) was 33.4%. A BMI of 30 to 35 was reported in 17.7%, while 15.7% had a BMI of more than 35 [12].

According to our PHQ-9 screening results, more than one-third of the participants suffered from moderate to severe depression. This result is higher than what was reported in a recent study in Saudi primary health care centers. They reported that 49.9% of the visitors exhibited depressive symptoms, 31% had mild, 13.4% had moderate, and 4.4% had severe depression [13].

Several meta-analysis studies were done to examine the relationship between depression and obesity using the WHO definition of obesity by calculating the BMI of the participants. One of the most critical findings in our research is that the majority of cases of depression were reported among underweight and obese individuals (43.4% and 41.7%), respectively. This U-shaped relationship is consistent with a previous study done in 2013 in the United Kingdom [14]. These exciting findings raise the awareness that underweight individuals are at high risk of developing depression.

Many studies were done to examine the relationship between obesity and depression due to their significant morbidity and mortality rate worldwide. Our research found that 23.5% of obese individuals had mild depressive symptoms compared to 31.6% with moderate to severe symptoms. A recent study in India reported similar findings. They recommended lifestyle modifications and mental health promotion programs as the primary intervention for obese people with a mild risk of developing depression [15]. They also recommended psychological counseling for obese participants with moderate to severe risk for depression.

This study showed a significant association between obesity, depression, and age. The association was more marked between those aged 18 to 25 years. They were at the highest risk of having both obesity and depression. A cross-sectional study conducted in Riyadh, Saudi Arabia reported a significant association between depression and obesity among young medical students [16]. Another cross-sectional study in Abha, Saudi Arabia showed similar results. Out of 389 participants, 71 were obese, and 31 (42.7%) of those obese participants presented with moderate to severe depression [17]. Moreover, A cross-sectional study was done in 2019 to measure the association between obesity and depression. The study concluded a significant association between these two variables and advanced age (p=0.008) [15]. Furthermore, another study was done in Al Kharj, Saudi Arabia, reported a significant association between obesity and psychological stress, which was more marked in participants above 30 [18]. In 2010, a meta-analysis included cross-sectional studies conducted to assess the correlation between depression and obesity and the influence of demographic factors on this association. A significant positive association was also found among adult women. However, they stated that this result was affected by the fact that there were not enough studies examining the older population [19]. Notably, our study’s results represented a strong association with younger age group. However, a study was conducted to analyze the responses to questionnaires, which stated that younger age groups attained more frequent responses than older age groups [20].

Our study showed a significant association between obesity and depression and being single. Out of 326 (45.9%), 42 (12.8%) single participants suffered from these comorbidities. A study in Abha, Saudi Arabia, done to measure this correlation in male students, showed that the correlation was more pronounced among single participants 381 (97.9%). However, this result may be because participants were mainly students, and marriage at this age is scarce [17]. In contrast to this study, another study done in Al Kharj, Saudi Arabia, showed no significant association between obesity, depression, and marital status (p = 0.210) [18].

Furthermore, our study showed a statistically significant association between obesity and the onset of depression. People who were obese for more than ten years were more likely to have depression. Xiang and An examined the association between obesity and the onset of depression among middle-aged and older adults by collecting data from 1994 to 2010. It was found that overweight and obese people were 13% and 9%, respectively, more likely to develop depression with 16 years of follow-up compared to people with normal weight [21]. However, another study investigated if obesity can cause depression with 12 years of follow-up. The result was that obesity at baseline did not significantly predict major depression episodes in women and negatively predicted major depression episodes in men [22]. According to our study, depressed and obese participants were more likely to have chronic diseases (17.6%). Further, we found that only 9.1% of obese and depressed people did not have any chronic disease.

According to Luck-Sikorski et al., the effect of double stigma from obesity and depression is significantly associated with a more negative attitude, on the depression stigma scale (p = 0.002) and on the fatphobia scale (p > 0.001), which in turn was associated with more negative health outcomes compared to single stigmatized disease [3]. Another study assessed the association between obesity and frequent mental distress with chronic diseases. The relationship was statistically significant (p > 0.0001) in six chronic diseases, which were diabetes, high blood pressure, coronary artery disease, stroke, asthma, and arthritis [23]. In primary health care centers in the United States, a study was performed to assess the relationship between race, obesity, depression, and chronic diseases. The association was significant (p < 0.05) between obesity, depression, diabetes, dyslipidemia, and hypertension [24].

Our study showed a strong relationship between obesity, depression, and psychiatric disorders. Around a fifth of obese depressed participants suffer from psychiatric disorders. On the other hand, 9.3% of people who had obesity and depressions were not diagnosed with psychiatric disorders. Other studies supported these findings. A cross-sectional study was conducted in a male secondary school in Saudi Arabia. The overall prevalence of mental disorders was anxiety (64.6%), depression (57%), and stress (39.4%), and there was a strong association between obesity, depression, anxiety, and stress (p >0.001 for all) [10]. According to a study done in the United States, obesity was associated with significant increases in lifetime diagnosis of major bipolar disorder, and panic disorder or agoraphobia. Those with a BMI score of 30 or more had a higher lifetime prevalence for mood disorders, including depression and anxiety than those with a BMI score of less than 30 [25]. Another study was conducted in Canada showed that obesity was positively associated with depression, panic attacks, mania, anxiety, social phobia disorder, and suicidal ideation. On the other hand, obesity and depression were negatively associated with drug dependence (95% CI 0.31-0.89). Although most of these results were more specific to women, some were also present in men [26].

We found that the group with depression and obesity is more likely to take medications regularly (18.3%) than those who don’t take regular medications (9.6%). This can be explained by the fact that antidepressant medications used by depressing patients lead to undesirable weight gain as a side effect [27]. The association between obesity and depression, in addition to the effect of depressive symptoms, was statically significant, around 22 (38.5%) participants suffer from moderate or severe symptoms that affect their daily life activity. In the United States, a cross-sectional study measures the association between waist circumference, abdominal obesity, and depression among overweight and obese adults. It was found that there is a strong association between waist circumference or abdominal obesity and major depressive disorder or moderate to severe depressive symptoms [28]. Another study was conducted in Egypt to measure the effects and the psychiatric morbidities in obese females. It showed an increased level of anxiety and depressive symptoms in obese patients, accounting for 58.5% and 46.3%, respectively [29].

By knowing the population at risk of developing depression, obesity, or both, we can emphasize prevention as well as mental and physical health promotion, especially among young adults in the community. This is important due to the high prevalence of obesity and the under-diagnosis of mental disorders such as depression. Having a high BMI of 30 or more was more common in moderate to severely depressed participants. Thus, preventing or controlling obesity will most likely improve mental health and vice versa.

Study limitations

The current study was conducted in a cross-sectional manner based on self-reporting questionnaires, which could have inaccurate results and response bias. Moreover, the results of the study cannot be generalized to a wider population, because it was conducted in one out of 13 provinces in Saudi Arabia. Besides, the diet was not included as a variable where it is an important factor for both obesity and depression. Further, extensive research on this topic in Saudi Arabia should be considered. This is because obesity and depression are major causes of morbidity and mortality. Therefore, they must be assessed and managed properly. Also, preventive and screening measures should be implemented.

## Conclusions

Depression and obesity are major health challenges worldwide. In Saudi Arabia, there is limited literature on this topic among the general population. This study was done to investigate the relationship between depression and obesity in adults living in the Eastern Province, Saudi Arabia with demographical data as variables. In order to find significant risk factors for developing depression, obesity, or both. The results showed a strong association between moderate to severe depression and obesity. The main findings of the most significant socioeconomic factors that associated with depression and obesity were in younger adults with age between 18 to 25 years (11.2%), being single (12.8%), having a BMI of 30 or more for 10 years or more (45.4%), the presence of associated chronic illnesses (17.6%), the presence of associated psychiatric disorder (18.3%) and intake of regular medication (18.3%). With these facts in mind, there is a need for health promotion campaigns targeting the high-risk young adult population to raise awareness about the dangerous outcome of depression and obesity on mental and physical health.

## Appendices

**Table 5:** Demographical data questionnaire

1. Nationality																
Saudi								Non-Saudi								
2. Gender																
Male								Female								
3. Age																
18 to 25		26 to 35				36 to 45				46 to 55					56 to 65	
4. Marital status																
Single				Married				Divorced					Widow			
5. Average monthly income																
Less than 5,000				5,000 to 10,000				11,000 to 15,000					More than 15,000			
6. Number of family members benefiting from the income																
1				2				3					More than 3 (specify)			
7. Educational level																
Elementary school				Middle school				High school					University or above			
8. Weight																
9. Height																
10. Do you suffer from any chronic illness?																
Hypertension	Diabetes				Asthma			Sickle cell disease				Hypothyroidism				Other (specify)
11. Do you suffer from any psychiatric illness?																
Obsessive compulsive disorder			Panic disorder				Schizophrenia				Depression			Other (specify)		
12. Do you take regular medications?																
No									Yes (specify):							

**Table 6:** Patient Health Questionnaire-9

PATIENT HEALTH QUESTIONNAIRE-9 (PHQ-9)				
Over the last 2 weeks, how often have you been bothered by any of the following problems? (Use “✔” to indicate your answer)	Not at all	Several days	More than half the days	Nearly every day
1. Little interest or pleasure in doing things	0	1	2	3
2. Feeling down, depressed, or hopeless	0	1	2	3
3. Trouble falling or staying asleep, or sleeping too much	0	1	2	3
4. Feeling tired or having little energy	0	1	2	3
5. Poor appetite or overeating	0	1	2	3
6. Feeling bad about yourself — or that you are a failure or have let yourself or your family down	0	1	2	3
7. Trouble concentrating on things, such as reading the newspaper or watching television	0	1	2	3
8. Moving or speaking so slowly that other people could have noticed? Or the opposite — being so fidgety or restless that you have been moving around a lot more than usual	0	1	2	3
9. Thoughts that you would be better off dead or of hurting yourself in some way	0	1	2	3
Total score				

Not difficult at all	Somewhat difficult	Very difficult	Extremely difficult

Informed consent to participate in a research study.

(The relationship between obesity and depression in adults, a cross-sectional study in the Eastern Province).

We thank you for your cooperation in this research study entitled obesity and depression in adults, a cross-sectional study in the Eastern Province. 

The objectives of this study include:

1- An estimation of the prevalence of depression and obesity among adults in the Kingdom of Saudi Arabia, specifically in the Eastern Province.

2- An investigation of demographic variables, including gender, education, and socioeconomic status, as a mediator influencing the relationship between depression and obesity.

3- Informing prospective researchers of the association between obesity and depression.

4- Knowing this relationship will help raise awareness about recognizing and preventing the negative health effects of both conditions.

Your participation will be by filling out the questionnaire.  Also, your participation in the study will be voluntary, and it does not entail any financial benefits, sample, or therapeutic benefits, and does not require any pharmacological or therapeutic interventions. You also have the right to refuse to participate in this study. We confirm that all the information that will be shared in this research study is subject to confidentiality, and the privacy and confidentiality of the participant will be considered, and it will be used exclusively for the purpose of the study. This study is designed for a possible benefit to you and to people like you. Also, the results of the study may be used in the future in alternative treatments.

I acknowledge my consent to participate in this research study, and my participation is voluntary and of my own will, and I give the research team the full right to act on the information I share, and I grant the research team the right to use this information for the purpose of other research studies in the future when needed, and without reference to me personally, whether the future study is related to the current study status or not.

Signature: ………………….

## References

[1] (2021). Depression. https://www.who.int/health-topics/depression.

[2] (2021). Obesity. https://www.who.int/topics/obesity/en/.

[3] Luck-Sikorski C, Schomerus G, Jochum T, Riedel-Heller SG (2018). Layered stigma? Co-occurring depression and obesity in the public eye. J Psychosom Res.

[4] Castanon N, Lasselin J, Capuron L (2014). Neuropsychiatric comorbidity in obesity: role of inflammatory processes. Front Endocrinol (Lausanne).

[5] van Belle G, Fisher LD, Heagerty PJ, Lumley T (2004). Biostatistics: A Methodology For the Health Sciences, 2nd Edition. https://www.wiley.com/en-us/Biostatistics%3A+A+Methodology+For+the+Health+Sciences%2C+2nd+Edition-p-9780471031857.

[6] Kroenke K, Spitzer RL, Williams JB (2001). The PHQ-9: validity of a brief depression severity measure. J Gen Intern Med.

[7] Daniel WW, Cross CL (2018). Biostatistics: A Foundation for Analysis in the Health Sciences. Sciences.

[8] Smith G (2021). Does gender influence online survey participation?: A record-linkage analysis of university faculty online survey response behavior. https://core.ac.uk/download/pdf/145691065.pdf.

[9] (2021). Demography Survey 2016. https://www.stats.gov.sa/sites/default/files/en-demographic-research-2016_2.pdf.

[10] Alshahrani M, Al Masoudi M, Alshahrani E (2021). Association between obesity and mental disorders among male secondary school students in Abha, Kingdom of Saudi Arabia: a predictor based cross-sectional study. Middle East Journal of Family Medicine.

[11] Al-Rethaiaa AS, Fahmy AE, Al-Shwaiyat NM (2010). Obesity and eating habits among college students in Saudi Arabia: a cross sectional study. Nutr J.

[12] Simon GE, Ludman EJ, Linde JA (2008). Association between obesity and depression in middle-aged women. Gen Hosp Psychiatry.

[13] Al-Qadhi W, Ur Rahman S, Ferwana MS, Abdulmajeed IA (2014). Adult depression screening in Saudi primary care: prevalence, instrument and cost. BMC Psychiatry.

[14] Carey M, Small H, Yoong SL, Boyes A, Bisquera A, Sanson-Fisher R (2014). Prevalence of comorbid depression and obesity in general practice: a cross-sectional survey. Br J Gen Pract.

[15] Garg R, Saxena SK, Bashir S (2019). Is obesity a risk to depression? A cross-sectional study. Ind Psychiatry J.

[16] Alrzoq Z, Aldayel F, Alrzoq M, Alrzoq R, Al-qumaizy K (2015). Depression and obesity among Saudi medical students using BDI-II at Imam Muhammad Ibn Saud Islamic University. International Academic Research Journal of Medical Sciences.

[17] AlQahtani A, Nahar S, AlAhmari SM, AlQahtani K (2015). Association between obesity and mental disorders among male students of King Khalid University, Abha. Saudi Arabia. Saudi Journal of Obesity.

[18] Aldossari KK, Shubair MM, Al-Ghamdi S (2021). The association between overweight/obesity and psychological distress: a population based cross-sectional study in Saudi Arabia. Saudi J Biol Sci.

[19] de Wit L, Luppino F, van Straten A, Penninx B, Zitman F, Cuijpers P (2010). Depression and obesity: a meta-analysis of community-based studies. Psychiatry Res.

[20] Smith MG, Witte M, Rocha S, Basner M (2019). Effectiveness of incentives and follow-up on increasing survey response rates and participation in field studies. BMC Med Res Methodol.

[21] Xiang X, An R (2015). Obesity and onset of depression among U.S. middle-aged and older adults. J Psychosom Res.

[22] Gariepy G, Wang J, Lesage AD, Schmitz N (2010). The longitudinal association from obesity to depression: results from the 12-year National Population Health Survey. Obesity (Silver Spring).

[23] Liu Y, Croft JB, Wheaton AG (2013). Association between perceived insufficient sleep, frequent mental distress, obesity and chronic diseases among US adults, 2009 behavioral risk factor surveillance system. BMC Public Health.

[24] Stecker T, Fortney JC, Steffick DE, Prajapati S (2006). The triple threat for chronic disease: obesity, race, and depression. Psychosomatics.

[25] Simon GE, Von Korff M, Saunders K, Miglioretti DL, Crane PK, van Belle G, Kessler RC (2006). Association between obesity and psychiatric disorders in the US adult population. Arch Gen Psychiatry.

[26] Mather AA, Cox BJ, Enns MW, Sareen J (2009). Associations of obesity with psychiatric disorders and suicidal behaviors in a nationally representative sample. J Psychosom Res.

[27] Faith MS, Butryn M, Wadden TA, Fabricatore A, Nguyen AM, Heymsfield SB (2011). Evidence for prospective associations among depression and obesity in population-based studies. Obes Rev.

[28] Zhao G, Ford ES, Li C, Tsai J, Dhingra S, Balluz LS (2011). Waist circumference, abdominal obesity, and depression among overweight and obese U.S. adults: National Health and Nutrition Examination Survey 2005-2006. BMC Psychiatry.

[29] Shalaby A, Sadik S, Mahmoud D (20206). Psychiatric morbidities of female obesity before and after dieting: an Egyptian sample. Middle East Current Psychiatry.

